# Modified Balloon Use After Rotational Atherectomy Reduces Major Adverse Cardiovascular Event Rates in Severely Calcified Coronary Lesions: A Systematic Review and Meta-Analysis

**DOI:** 10.3390/jcm13226853

**Published:** 2024-11-14

**Authors:** Réka Ehrenberger, Richárd Masszi, Előd-János Zsigmond, Uyen Nguyen Do To, Caner Turan, Anna Walter, Péter Hegyi, Marie Anne Engh, Gábor Zoltán Duray, Zsolt Molnár, Béla Merkely, István Ferenc Édes

**Affiliations:** 1 Centre for Translational Medicine, Semmelweis University, 1085 Budapest, Hungary; reka.ehrenberger@gmail.com (R.E.);; 2 Heart and Vascular Centre, Semmelweis University, 1122 Budapest, Hungary; 3 Department of Cardiology, Central Hospital of Northern Pest, Military Hospital, 1134 Budapest, Hungary; 4 Doctoral School of Clinical Medicine, University of Szeged, 6720 Szeged, Hungary; 5 Department of Anaesthesiology and Intensive Therapy, Semmelweis University, 1428 Budapest, Hungary; 6 Institute for Translational Medicine, Medical School, University of Pécs, 7624 Pécs, Hungary; 7 Institute of Pancreatic Diseases, Semmelweis University, 1083 Budapest, Hungary; 8 Department of Anaesthesiology and Intensive Therapy, Poznan University of Medical Sciences, 61-701 Poznan, Poland

**Keywords:** calcified coronary artery disease, plaque modification, rotational atherectomy, modified balloon, major adverse cardiovascular events

## Abstract

**Background/Objectives:** Calcified coronary lesions require plaque modification techniques for optimal stent apposition, of which rotational atherectomy (RA) is the most commonly used one. Challenging cases require the use of additional dedicated devices (such as modified balloons, MB); however, data available for evidence-based device selection are limited. The aim of this study is to determine the impact of the balloon-based technology used after successful RA treatment on outcomes. **Methods:** This study was carried out according to the PRISMA guidelines. MEDLINE, CENTRAL and Embase databases were systematically searched for eligible randomized and non-randomized studies. **Results:** A total of nine studies and 1024 patients were included in the analysis. Patients were treated with RA followed by either plain balloon angioplasty (RA + BA) or modified balloon (RA + MB) treatment prior to stent implantation. There was no significant difference in MACE (major adverse cardiovascular events; OR: 0.53; 95% CI: 0.21–1.34; *p* = 0.153), all-cause mortality (OR: 0.68; 95% CI: 0.33–1.42; *p* = 0.265), and target lesion revascularization (OR: 0.64; 95% CI: 0.27–1.55; *p* = 0.264) between the two groups. However, a sensitivity analysis demonstrated a significant decrease in MACE for patients with severely calcified lesions (OR: 0.42; 95% CI: 0.25–0.70; *p* = 0.009) in the RA + MB group. The analyses of the safety outcomes of slow flow/no reflow (OR: 0.59; 95% CI: 0.29–1.22; *p* = 0.128) and coronary artery perforation (OR: 1.18; 95% CI: 0.70–1.99; *p* = 0.480) showed no difference between the two groups. **Conclusions:** Our meta-analysis suggests that the benefit of the more invasive RA + MB treatment is statistically significant for severely calcified lesions, but is not associated with additional procedural complications.

## 1. Introduction

Despite advancements in treatment and prevention, ischemic heart disease remains a challenge in modern medicine with significant implications for public health and clinical practice. This necessitates the continuous development of an ever-expanding toolkit of interventional cardiology. These innovations have made it possible to treat complex anatomical and pathological conditions with initially poor prognoses, such as heavily calcified coronary artery disease.

Studies have shown that complete revascularization of heavily calcified lesions is less likely to be feasible and have demonstrated a correlation between high calcium burden and higher rates of postoperative mortality and overall adverse events [1,2,3]. Patients with moderate to severe calcification may have a 62% higher chance of developing stent thrombosis, whereas the risk of target lesion revascularization (TLR) has been shown to increase by 44% compared to non-calcified lesions [3]. The overall rate of major adverse cardiovascular events (MACEs) in this high-risk patient group can be as high as 30% [4].

Coronary lesions with extensive calcification and resistant plaque burden require preparation by thorough plaque modification for optimal stent apposition [5,6,7]. Rotational atherectomy (RA), a well-established device for this purpose, works by directly removing the calcified mass, preparing the coronary wall for stent implantation, which results in a reduction in the high complication rate in the era of drug-eluting stents as proven by numerous studies [3,8,9]. In the presence of calcified coronary arteries, the combination of rotational atherectomy (RA) with plain balloons, commonly referred to as workhorse devices, has demonstrated enhanced effectiveness compared to RA alone; therefore, there is consensus on implementing balloon-based devices after RA for optimal stent dilatation [10,11]. Additionally, the benefits of modified balloon techniques have been demonstrated in the PREPARE-CALC trial [12], with favorable long-term clinical outcomes. Previous clinical observations indicate that a plaque modification approach involving rotational atherectomy and modified balloons is associated with enhanced cross-sectional area (CSA) gain and optimal stent expansion during PCI [13]. Furthermore, existing evidence suggests that a suboptimal stent expansion predicts an increased risk of short- and long-term adverse events [14,15]. The current understanding is that aggressive balloon use facilitated by RA shows its genuine impact in highly calcified cases, resulting in a reduction in the high complication rates [16,17]. Nonetheless, the definitions of heavily calcified lesions seem more consistent across the reviewed manuscripts and interventional care. These include uncrossable and undilatable lesions, in which the use of RA-based aggressive balloon modification techniques is mandatory [7]. Nevertheless, the existing evidence on this matter is considerably limited, and the outcomes are frequently inconclusive.

Recently, Patel et al. [18] conducted a meta-analysis on the impact of cutting balloon dilatation after rotational atherectomy on periprocedural and long-term outcomes, but they could not demonstrate its significant effect. Given the importance of the topic and the increasing amount of data, we opted for a broader perspective and subgroup analyses to generate a clearer consensus in clinical practice.

Our aim was to investigate whether the treatment of moderately and severely calcified coronary arteries using a combination of rotational atherectomy and modified balloon techniques provides better short-, medium-, and long-term outcomes than rotational atherectomy combined with conventional balloon types.

## 2. Materials and Methods

We report our systematic review and meta-analysis, strictly following the PRISMA 2020 guidelines [19] (Table S1) and the Cochrane Handbook [20]. The protocol of the study was registered on PROSPERO (registration number CRD42022375595) and followed strictly, complemented by non-pre-specified post hoc analyses.

No ethical approval was required, as all data analyzed are publicly available, and no patients were involved in the design, conduct, or interpretation of our study.

### 2.1. Eligibility Criteria

Our analysis included studies reporting on patients with moderately or severely calcified coronary artery lesions treated with rotational atherectomy combined with either plain balloon angioplasty (RA + BA) or dedicated modified balloon types (RA + MB), in particular cutting or scoring balloons, prior to drug-eluting stent implantation.

Randomized and non-randomized comparative clinical studies met the inclusion criteria with the following requirements: (1) studies involving patients above 18 years of age with moderately or severely calcified coronary plaques, regardless of the evaluation method of calcification; (2) a comparison of rotational atherectomy combined with cutting balloon, scoring balloon or plain balloon; (3) reported event rates with a sample size of all-cause mortality, acute coronary syndrome (ACS), target lesion/vessel revascularization (TLR/TVR), target vessel failure (TVF), in-stent restenosis (ISR), stent thrombosis, slow flow/no reflow, and coronary artery perforation.

Studies reporting cases with only chronic total occlusion, in-stent restenosis (ISR), or without stent implantation were excluded. Data reported only in conference abstracts were not included in the analysis.

### 2.2. Information Sources

Our systematic search was conducted on the 14th of November 2022 and was revised on the 23rd of August 2024 in the following databases: MEDLINE (via PubMed), Embase, and CENTRAL (the Cochrane Central Register of Controlled Trials).

### 2.3. Search Strategy

During the systematic search, the following search keys were used: “calcification”, “coronary”, “percutaneous coronary intervention”, “atherectomy”, and “balloon angioplasty”. The detailed search strategy is available in the Supplementary Materials.

### 2.4. Selection Process

The articles retrieved with our search strategy were imported into EndNote for further screening and automatic and manual duplicate removal. The selection process was carried out independently by three review authors (R.E., E.-J.Zs., and R.M.), based on a predefined selection protocol. Interrater agreement was quantified by calculating Cohen’s kappa.

Articles in Chinese were translated using online tools, including Google Translate and Baidu Translate, to facilitate inclusion in the meta-analysis.

### 2.5. Data Collection Process

Two authors (R.E. and U.N.D.T.) independently collected data from the eligible articles using identical pre-structured tables.

The following data were extracted: first author, year of publication, study population, study period, duration of follow-up, baseline demographic characteristics, comorbidities, lesion and intervention characteristics, and outcome measures for the two treatment groups separately.

To mitigate heterogeneity due to diverse follow-up durations, we selectively extracted and analyzed data from similar time periods whenever feasible.

### 2.6. Study of Risk of Bias and Certainty of Evidence Assessment

Two authors (R.E. and U.N.D.T.) independently assessed the risk of bias using the Cochrane risk of bias assessment tools: the Risk Of Bias In Non-Randomized Studies of Interventions (ROBINS-I) [21] for non-randomized studies and the Risk of Bias 2 (RoB 2) [22] tool for randomized studies. The risk of bias for the individual studies and each outcome was assessed based on the recommendation of the Cochrane Collaboration. An independent third investigator (C.T.) resolved the arising disagreements.

The certainty of evidence assessment in the included studies was performed with GRADE-Pro [23] by two independent authors (R.E. and U.N.D.T.), based on the recommendations of the Cochrane Collaboration [20].

### 2.7. Synthesis Methods

Due to the substantial differences in MACE definitions used in the included articles, we recalculated MACEs for each one to improve comparability across studies, using the following components (if available): all-cause mortality, acute coronary syndrome (ACS), target lesion revascularization (TLR), target vessel revascularization (TVR), target vessel failure (TVF), in-stent restenosis (ISR), and stent thrombosis.

For the purposes of the meta-analysis, randomized trials and non-randomized studies were separately grouped with studies of the same design. The resulting analyses are represented by forest plots showing the subgroups and the overall pooled analysis as well.

The statistical analyses were performed using R [24] (version 4.1.2) with the meta (version 6.1.0) package for calculations and plots. Data from the selected studies were summarized using the Mantel–Haenszel method [25]. Pooled odds ratios (ORs) with 95% confidence intervals (CIs) were calculated using two-by-two tables. A random-effects meta-analysis model with the Hartung–Knapp [26] adjustment was used for each analysis. Forest plots were used to display pooled and individual study results. A *p*-value threshold of <0.05 was considered statistically significant in our analysis. The 95% prediction interval was used following the recommendations of IntHout et al. [27].

Statistical heterogeneity was evaluated using the I^2^ and χ^2^ tests [28], with a *p*-value threshold of <0.1, indicating a significant difference. Overall heterogeneity contribution and the influence of each effect size were visualized by Baujat plots [29].

## 3. Results

### 3.1. Study Search and Selection

**Figure 1:**
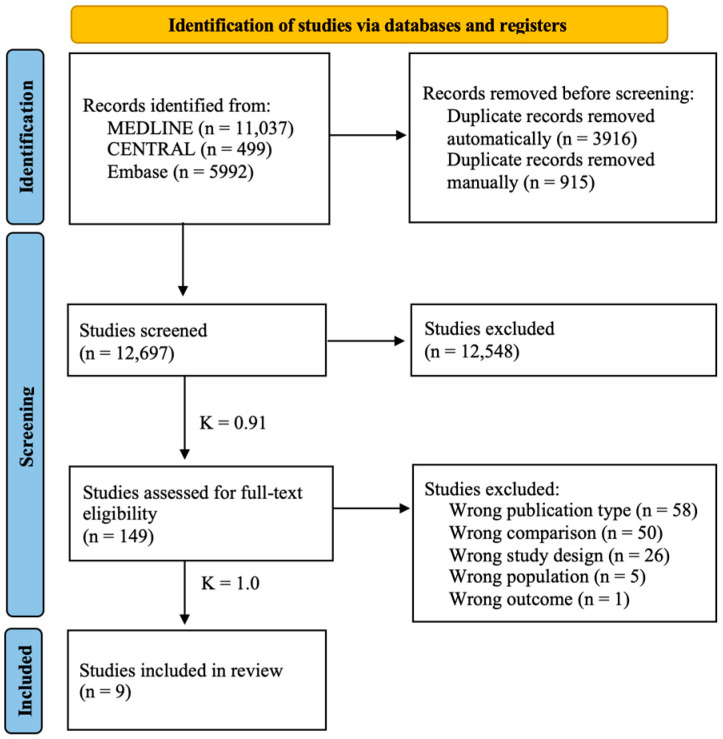
**PRISMA flowchart of study selection**. Selection process represented by the Preferred Reporting Items for Systematic Reviews and Meta-Analyses diagram. K = Cohen’s kappa coefficient.

The database search yielded 17,528 publications. After duplicate removal, 12,697 articles were included for title and abstract screening, of which 149 were deemed eligible for full-text review. After a thorough evaluation, nine articles [13,16,30,31,32,33,34,35,36] met our eligibility criteria for inclusion in the meta-analysis. The selection process and the reasons for exclusion are specified in the PRISMA flowchart (Figure 1).

### 3.2. Basic Characteristics of Studies Included

Among the eight studies included, six were observational [13,16,30,31,33,35], and three were randomized controlled trials [32,34,36]. Three studies were conducted in Japan, four in China, one in the USA, and one in Germany, with publication dates between 2012 and 2024. The final analysis included data from a total of 1024 patients.

The summary of baseline and intervention characteristics for each study is presented in Table 1 and Table S2.

All studies evaluated the efficacy of rotational atherectomy followed by modified balloon or plain balloon angioplasty for plaque modification prior to stent implantation in moderately or severely calcified coronary lesions. The modified balloons used in the included studies were cutting and scoring balloons. Of the studies included in the meta-analysis, seven used cutting balloons and two used scoring balloons.

The assessment of calcification varied between studies, with most relying solely on angiographic evaluation, whereas two utilized intravascular-ultrasound (IVUS) guidance [30,34]. All studies reported complete revascularization, defined as achieving residual stenosis < 30% and thrombolysis in myocardial infarction (TIMI) flow grade 3 post-procedure. Data from intravascular imaging techniques such as IVUS [13,31,32,33,34,36] and optical coherence tomography (OCT) [35] were used to assess the efficacy of blood-flow restoration and stent expansion. Procedural success was evaluated by coronarography and quantitative coronary angiography (QCA).

All included studies that reported on medication regimens indicated the use of dual antiplatelet therapy and anticoagulation in the procedure.

The study encompassed a range of follow-up periods from 1 to 36 months, allowing for a comprehensive evaluation of the short-, medium-, and long-term outcomes.

### 3.3. Major Adverse Cardiovascular Events (MACEs)

**Figure 2:**
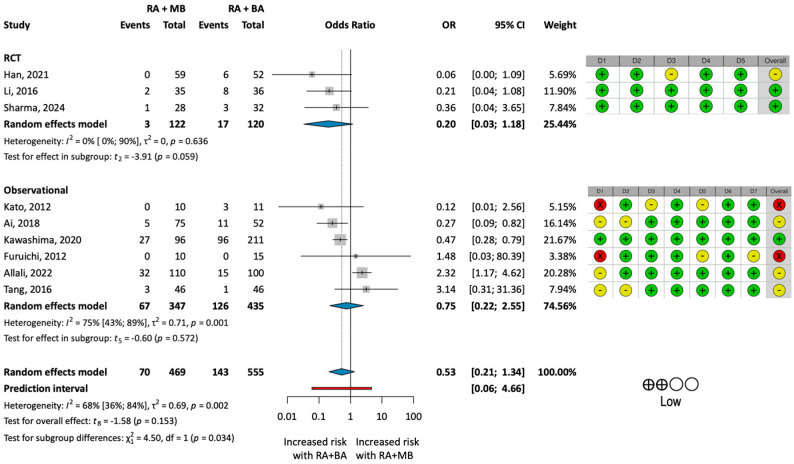
**Results of the analysis of MACE rates.** (**Left**): forest plot presenting the analysis of major adverse cardiovascular event (MACE) rate for the two groups treated with rotational atherectomy combined with either modified balloon types (RA + MB) or with plain balloon angioplasty (RA + BA). (**Right**): results of the risk of bias and certainty of evidence assessment. Green indicates low risk, yellow indicates moderate risk, and red indicates serious risk of bias. BA = plain balloon angioplasty; MB = modified balloon; RA = rotational atherectomy [13,16,30,31,32,33,34,35,36].

Our primary outcome of interest was the rate of MACEs as a composite outcome (Figure 2).

The pooled analysis of all studies could not detect any statistical difference between the MACE rates of the two groups (OR: 0.53; 95% CI: 0.21–1.34; *p* = 0.153).

The pooled analysis for each component of MACEs showed no significant difference between the groups in terms of ACS (OR: 1.16; 95% CI: 0.75–1.79; *p* = 0.456), all-cause mortality (OR: 0.68; 95% CI: 0.33–1.42; *p* = 0.265), TLR (OR: 0.64; 95% CI: 0.27–1.55; *p* = 0.264), TVR (OR: 0.79; 95% CI: 0.37–1.70; *p* = 0.461), or stent thrombosis (OR: 0.93; 95% CI: 0.49–1.77; *p* = 0.802).

The detailed results and forest plots of the additional analyses are included in the Supplementary Materials (Figures S1–S5).

A meta-analysis could not be performed for the outcomes of ISR and TVF due to the lack of reported outcomes in the included studies.

#### Omitting the Outlier

**Figure 3:**
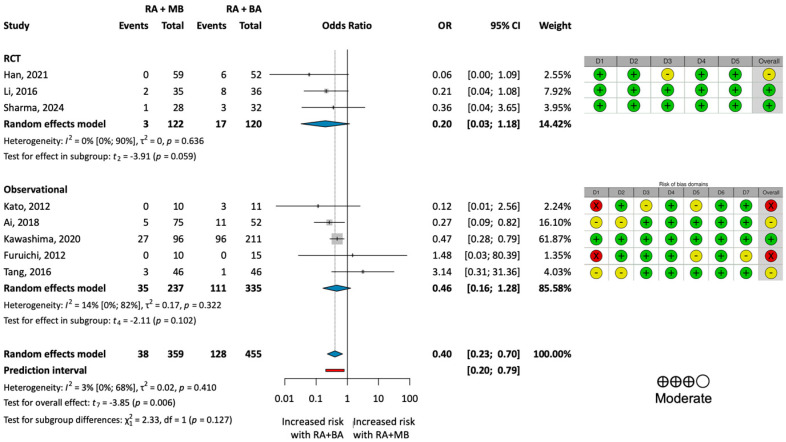
**Results of the analysis of MACE rates excluding the outlier.** (**Left**): forest plot representing the analysis of the MACE rate for the two treatment groups omitting Allali et al. [35]. (**Right**): results of the risk of bias and certainty of evidence assessment. Green indicates low risk, yellow indicates moderate risk, and red indicates serious risk of bias. BA = plain balloon angioplasty; MB = modified balloon; RA = rotational atherectomy [13,16,30,31,32,33,34,36].

The Baujat plot (Figure S6) revealed that the substantial heterogeneity (I^2^ = 68%; *p* = 0.002) observed in the analysis of MACEs was primarily due to a single study, published by Allali et al. [35]. Upon closer examination of the paper, we found that the significantly more severe calcification observed in the intervention group may have contributed to the higher complication rates. This analysis of the pooled data omitting the study by Allali et al. resulted in an OR of 0.40 (95% CI: 0.23–0.70; *p* = 0.006), indicating a statistically significant reduction in the MACE rate in the RA + MB group (Figure 3).

### 3.4. Procedural Outcomes

In the analysis of the special procedural outcome, slow flow/no reflow resulted in an odds ratio of 0.59 (95% CI: 0.29–1.22; *p* = 0.128), showing no significant difference between the two treatment groups. Similarly, the odds ratio for the rate of coronary perforation between the groups was 1.18 (95% CI: 0.70–1.99; *p* = 0.480), indicating no significant difference. These findings provide compelling evidence to support the safety of the more aggressive RA + MB approach. The forest plots for these analyses are presented in Figure S7.

### 3.5. Severely Calcified Coronary Lesions

**Figure 4:**
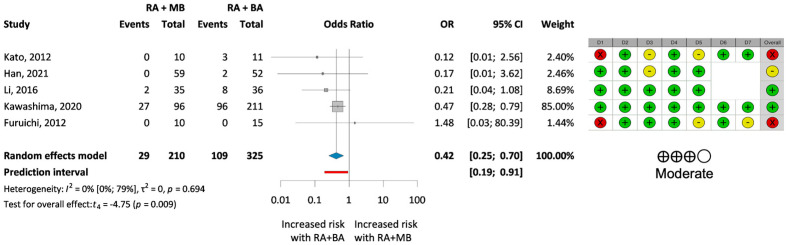
**Results of the analysis of MACE rates in severely calcified cases.** (**Left**): forest plot presenting the analysis of MACE rate in the two treatment groups for severely calcified coronary lesions. (**Right**): results of the risk of bias and certainty of evidence assessment. Green indicates low risk, yellow indicates moderate risk, and red indicates serious risk of bias. BA = plain balloon angioplasty; MB = modified balloon; RA = rotational atherectomy [13,16,30,32,34].

A subgroup analysis of severely calcified coronary lesions included five studies [13,16,30,32,34] with 535 patients (Figure 4). A statistically significant reduction in the MACE rate with OR of 0.42 (OR 0.42; 95% CI 0.25–0.70; *p* = 0.009) was observed in those treated with modified balloons instead of plain balloons after rotational atherectomy.

### 3.6. Risk of Bias Assessment and Publication Bias

The risk of bias assessment revealed some concerns about the quality of the studies included in the analysis, particularly about possible confounders, as most studies were not randomized.

Additional tables and funnel plots with the results of the risk of bias assessment and publication bias can be found in the Supplementary Materials (Figures S8–S10).

### 3.7. Level of Evidence

The certainty of evidence was low for most outcomes in our analysis. The downgrading of the evidence was a consequence of the marked heterogeneity in the MACE outcome, the high risk of bias, and the low number of event rates in the remaining outcomes (Table S3).

## 4. Discussion

Calcified coronary artery disease remains a problematic burden for coronary intervention specialists. Rotational atherectomy, as a major method of clearing lesions for further preparation, has been a hallmark of treatment for more than 30 years [37,38,39,40,41]. However, the device selection after successful RA to achieve optimal results remains to be understood.

Our analysis of the available literature sought to determine which balloon-based additional manipulation methods seemed optimal after RA to achieve sufficient clinical results following PCI.

In assessing the comparative effectiveness of modified versus plain balloons post-RA, our findings align partially with the existing body of literature. Our pooled analysis found no significant improvements in MACEs, all-cause mortality, TVR, TLR, or stent thrombosis, consistent with previous observations (Kato et al. [30], Han et al. [34], and Li et al. [32]). However, these studies highlighted notable benefits of using modified balloons after RA treatment, underscoring the potential clinical relevance despite the lack of statistical significance. The lack of differential outcomes might be attributed to several factors, including the variability in lesion characteristics, operator experience, and procedural techniques across the studies analyzed.

To reduce heterogeneity due to definition, our uniform definition of MACEs was chosen to encompass all clinically relevant events that burden both the patient and the healthcare provider. The examination of MACE rates revealed that the RA + MB group experienced nearly half as many clinical events as the RA + BA group. This finding is clinically relevant and aligns with our initial hypothesis, yet it did not achieve statistical significance. This may be due to the fact that some of the studies [31,33,35,36] involved PCI with both moderately and severely calcified lesions. The unequal distribution of these lesions within the two groups stems from the non-randomized methodology employed in the majority of the included studies, potentially contributing to the significant degree of heterogeneity in the study population.

The high degree of heterogeneity in the summation information was mainly due to one study (Allali et al. [35]), where the RA + MB group had significantly more severely calcified cases compared to the RA + BA group, resulting in higher rates of adverse events. Notably, this marked difference in disease severity was not observed across the other included studies. To address this issue, we conducted a separate analysis omitting this outlier, which reduced heterogeneity. The results proved statistically significant, demonstrating a reduction in MACE rates in the RA + MB group in a comparable patient population.

Moreover, the subgroup analysis focusing on severely calcified lesions revealed a statistically significant reduction in MACE rates for the RA + MB group compared to the control group.

The significant reduction in MACEs observed in our outlier-adjusted and subgroup analyses challenges some earlier findings and underscores the potential of modified balloons in more complex coronary scenarios. This aligns with the findings of Kawashima et al. [16], who also noted better outcomes in such subgroups. This outcome indicates that a stratified approach is necessary, where the choice of balloon type is tailored based on specific lesion characteristics rather than a one-size-fits-all approach. The findings support the consensus that adequate plaque modification is mandatory in the treatment of coronary disease [6,7], although as a novelty, it highlights the importance of multiple, sequential lesion preparation devices in this scenario with the aim of reducing MACE rates.

The slow-flow/no-reflow phenomenon and coronary perforation are serious but preventable complications of RA-based revascularization. These complications were infrequent and, based on our findings, were generally associated with the RA procedure itself rather than the type of balloon used, emphasizing that lesion characteristics and procedural complexity likely play a more prominent role in influencing these outcomes. The incidence of the former has decreased from 15% to 0–2.6% [4,7,38,42], whereas for the latter, recent studies suggest a range between 0% and 2.0% [4,7,39,42], which still raises the question of the relationship between the occurrence and the device of choice. The results indicated no significant difference between the two groups, which supports the safety of the combination of the dedicated RA + MB methods when performed with care, despite its more aggressive nature. Coronary dissection is a critical consideration, particularly in light of the findings of Sharma et al. [36]. This research revealed a high prevalence of dissections on IVUS in both investigated groups. Notably, most dissections were confined to the intima, with none extending into the media. Furthermore, after stent implantation, the final IVUS assessment showed a decrease in the number of observed dissections. These results indicate that while dissections are common during plaque modification, they are primarily superficial and may not lead to significant complications if properly managed. Therefore, vigilant monitoring during and after these interventions remains essential.

This meta-analysis is the most comprehensive analysis of the topic to date, adhering to the latest guidelines with rigorous methodology, thereby ensuring the utmost quality, transparency, and reproducibility of the results. One of the notable strengths of the research is the use of standardized definitions of endpoints and the recalculation of existing data to establish greater coherence in our results. Additionally, sensitivity analyses were conducted to increase the reliability of our findings.

In terms of limitations, the most important ones are the low number of events and the limited number of RCTs on the topic; thus, most of the included studies were observational studies, introducing a high risk of confounding factors, aligned with the lack of justification for the choice of treatment, which could potentially introduce bias. We have to emphasize the inconsistency in the follow-up times across the included studies, which may have influenced the results despite the adjustments described in Section 2. Furthermore, the evaluation of calcification also varied between studies. These differences may affect the distinction between severe and moderate cases. Another limitation of our meta-analysis is the restricted ability to compare different types of modified balloons. Most of the included studies focused on cutting balloons, with only a few using scoring balloons, which limits meaningful comparison between balloon types.

The results of our study provide guidance for the treatment of coronary artery disease with severe calcification. While patient-specific risk factors and operator expertise should guide the choice of balloon type, the use of modified balloon types after RA may be beneficial for patients with highly calcified lesions by reducing the rate of MACEs associated with the intervention, whereas plain balloons may be sufficient for less severe calcifications. However, it is crucial for clinicians to assess lesion severity not only through angiographic evaluation but also by employing advanced imaging techniques such as intravascular ultrasound (IVUS) or optical coherence tomography (OCT) when available. This comprehensive approach to lesion assessment will enable more informed clinical decision-making tailored to individual patient needs.

Future investigations should explore the role of modified balloons in treating various lesion types, including those with different calcification patterns or morphologies. We recommend that future studies conduct a more comprehensive evaluation of various balloon types in the context of RA with an emphasis on assessing long-term outcomes. Future investigations should also evaluate how patient-specific factors influence the efficacy of these interventions, including anatomical considerations and comorbid conditions. Coronary artery calcification has a multifactorial pathology; therefore, all emerging co-factors and confounders should be excluded by randomization or adjusted in future investigations, including comorbidities and risk factors such as hypertension, hyperlipidemia, diabetes mellitus, and smoking, as well as the location and severity of calcification (moderate or severe). As for the latter, standard and objective evaluation of lesion characteristics using intravascular imaging techniques is mandatory for consistent results. Insufficient data on the relationship between mortality, recurrent acute coronary syndrome, repeated revascularization, and the type of intervention require further research to reach a clear consensus and formulate guidelines.

## 5. Conclusions

Our meta-analysis suggests that highly invasive RA + MB treatment is most beneficial for truly severe calcified lesions, while not increasing the risk of procedural complications. These findings provide evidence for the overall safety and efficacy of this approach.

## Figures and Tables

**Table 1:** Baseline characteristics of studies included.

Author, Year	Study Site (Number of Centers)	Study Design	Number of Patients (Male%)	RA + MB (*n*)	RA + BA (*n*)	Type of Modified Balloon	Age (Year)		Follow-Up Period (Months)	Severe Calcification (%)
							RA + MB	RA + BA		
Furuichi, 2012 [13]	Japan (1)	Retrospective observational	25 (ND)	10	15	cutting	ND	ND	6	100/100
Kato, 2012 [30]	Japan (1)	Retrospective observational	21 (48)	10	11	scoring	76 ± 5	70 ± 13	12 ± 5	100/100
Tang, 2016 [31]	China (1)	Retrospective observational	92 (58)	46	46	cutting	66 ± 10	70 ± 7	9	67.4/76.1
Li, 2016 [32]	China (2)	RCT	71 (70)	35	36	cutting	69.3 ± 11.6	72.2 ± 10.2	13.2 ± 4.7	100/100
Ai, 2018 [33]	China (1)	Retrospective observational	127 (76)	75	52	cutting	66.1 ± 8.8	64.7 ± 8.4	13.5 (8.5–26.8)	78.7/90.4
Kawashima, 2020 [16]	Japan (3)	Retrospective observational	307 (69)	96	211	scoring	72.0 (68.0–77.3)	72 (63–77)	36	100/100
Han, 2021 [34]	China (1)	RCT	120 (66)	60	60	cutting	70.8 ± 8.3	71.7 ± 9.3	12	100/100
Allali, 2022 [35]	Germany (1)	Prospective observational	210 (78)	110	100	cutting	74.9 ± 8.2	74.8 ± 7.1	9	94.3/76.5 *
Sharma, 2024 [36]	USA (2)	RCT	60 (78)	29	31	cutting	69.2 ± 10	72.8 ± 8.7	1	72.4/74.2

Parameters presented as exact numbers, mean with standard deviation, or median with IQR (first and third quartile). BA = plain balloon angioplasty; MB = modified balloon; ND = no data available; RA = rotational atherectomy; RCT = randomized controlled trial. * Indicates significant differences between the groups.

## Data Availability

The datasets used in this study can be found in the full-text articles included in the systematic review and meta-analysis.

## Supplementary Materials

The following supporting information can be downloaded at: https://www.mdpi.com/article/10.3390/jcm13226853/s1, Search strategy, Figure S1: Results of the analysis of all-cause mortality rates; Figure S2: Results of the analysis of TLR rates; Figure S3: Results of the analysis of ACS rates; Figure S4: Results of the analysis of stent thrombosis rates; Figure S5: Results of the analysis of TVR rates; Figure S6: Baujat plot; Figure S7: Results of the analyses of safety outcomes; Figure S8: Risk of bias assessment for MACE using RoB2; Figure S9: Risk of bias assessment for MACE using ROBINS-I; Figure S10: Funnel plot for publication bias; Table S1: PRISMA 2020 checklist; Table S2: Extended baseline and intervention characteristics; Table S3: Certainty of evidence assessment of the individual outcomes using GRADE-Pro.
