# Supplementary material for: Modified Balloon Use After Rotational Atherectomy Reduces Major Adverse Cardiovascular Event Rates in Severely Calcified Coronary Lesions: A Systematic Review and Meta-Analysis
Source: J Clin Med. 2024 Nov 14;13(22):6853. doi: 10.3390/jcm13226853 (PMC11594699; doi:10.3390/jcm13226853)
Supplement: Supplementary file 1 [file jcm-13-06853-s001.zip › jcm-3275166-supplementary.pdf]

SUPPLEMENTARY MATERIAL

Modified Balloon Use After Rotational Atherectomy Reduces Major Adverse Cardiovascular Event Rates in Severely Calcified Coronary Lesions: A Systematic Review and Meta-Analysis

AUTHORS

Réka Ehrenberger [1,2], Richárd Masszi [1,2], Előd-János Zsigmond [1,3,4], Uyen Nguyen Do To [1], Caner Turan [1,5], Anna Walter [6], Péter Hegyi [1,6,7], Marie Anne Engh [1], Gábor Zoltán Duray [1,2,3], Zsolt Molnár [1,5,8], Béla Merkely [2] and István Ferenc Édes [2,*]

Affiliations:

1 Centre for Translational Medicine, Semmelweis University, 1085 Budapest, Hungary; reka.ehrenberger@gmail.com (R.E.); masszi.richard@gmail.com (R.M.); zsigmond_elod@yahoo.de (E.-J.Z.); touyenpro127@gmail.com (U.N.D.T.); c.caner.turan@gmail.com (C.T.); hegyi2009@gmail.com (P.H.); marieaengh@gmail.com (M.A.E.); gduray@yahoo.com (G.D.); zsoltmolna@gmail.com (Z.M.)
2 Heart and Vascular Centre, Semmelweis University, 1122 Budapest, Hungary; merkely.bela@gmail.com
3 Department of Cardiology, Central Hospital of Northern Pest, Military Hospital, 1134 Budapest, Hun-gary
4 Doctoral School of Clinical Medicine, University of Szeged, 6720 Szeged, Hungary
5 Department of Anaesthesiology and Intensive Therapy, Semmelweis University, 1428 Budapest, Hun-gary
6 Institute for Translational Medicine, Medical School, University of Pécs, 7624 Pécs, Hungary; walteranna.tm@gmail.com
7 Institute of Pancreatic Diseases, Semmelweis University; 1083 Budapest, Hungary
8 Department of Anaesthesiology and Intensive Therapy, Poznan University of Medical Sciences; 61-701 Poznan, Poland
* Correspondence: edes789@gmail.com; Tel.: +(36)-20-663-2488

Address for correspondence

István Ferenc Édes MD, PhD, FESC

E-mail address: edes789@gmail.com

TABLE OF CONTENTS

Supplementary Methods. Search strategy **3**

Supplementary Figure S1. Results of the analysis of all-cause mortality rates **4**

Supplementary Figure S2. Result of the analysis of TLR rates **4**

Supplementary Figure S3. Results of the analysis of ACS rates **5**

Supplementary Figure S4. Results of the analysis of stent thrombosis rates **5**

Supplementary Figure S5. Results of the analysis of TVR rates **6**

Supplementary Figure S6. Baujat plot **6**

Supplementary Figure S7. Results of the analyses of safety outcomes **7**

Supplementary Figure S8. Risk of bias assessment for MACE using RoB2 **8**

Supplementary Figure S9. Risk of bias assessment for MACE using ROBINS-I **8**

Supplementary Figure S10. Funnel plot for publication bias **9**

Supplementary Table S1. PRISMA 2020 checklist **10**

Supplementary Table S2. Extended baseline and intervention characteristics **13**

Supplementary Table S3. Certainty of evidence assessment of the individual outcomes using GRADE-Pro **14**

1 Supplementary Methods

SEARCH STRATEGY

MEDLINE (via Pubmed): ("calcif*"[All Fields] OR "LHCC"[All Fields]) AND ("coronaries"[All Fields] OR "heart"[MeSH Terms] OR "heart"[All Fields] OR "coronary"[All Fields] OR ("coronaries"[All Fields] OR "heart"[MeSH Terms] OR "heart"[All Fields] OR "coronary"[All Fields]) OR ("heart"[MeSH Terms] OR "heart"[All Fields] OR "hearts"[All Fields] OR "heart s"[All Fields])) AND ("PCI"[All Fields] OR "Percutaneous Coronary Intervention"[All Fields] OR ("percutaneous"[All Fields] OR "percutaneously"[All Fields] OR "percutanous"[All Fields]) OR ("percutaneous"[All Fields] OR "percutaneously"[All Fields] OR "percutanous"[All Fields]) OR ("percutaneous"[All Fields] OR "percutaneously"[All Fields]) OR ("modification"[All Fields] OR "modifications"[All Fields]) OR ("atherectomy"[MeSH Terms] OR "atherectomy"[All Fields] OR "atherectomies"[All Fields]) OR ("angioplastied"[All Fields] OR "angioplasty"[MeSH Terms] OR "angioplasty"[All Fields] OR "angioplasties"[All Fields]) OR ("balloon"[All Fields] OR "balloon s"[All Fields] OR "balloons"[All Fields]))

Embase: (calcif* OR lhcc) AND (coronary OR coronaries OR 'heart'/exp OR heart) AND ('pci' OR 'percutaneous coronary intervention'/exp OR 'percutaneous coronary intervention' OR percutaneous OR percutaneously OR 'modification'/exp OR modification OR 'atherectomy'/exp OR atherectomy OR 'angioplasty'/exp OR angioplasty OR 'balloon'/exp OR balloon)

CENTRAL: (calcif* OR LHCC) AND (coronary OR coronaries OR heart) AND ("PCI" OR "Percutaneous Coronary Intervention" OR percutaneous OR percutaneously OR modification OR atherectomy OR angioplasty OR balloon)

3 Supplementary Figures

Supplementary Figure S1. **Results of the analysis of all-cause mortality rates.** Forest plots presenting the analysis of the all-cause mortality rate for the two groups treated with rotational atherectomy combined with either modified balloon types (RA+MB) or with plain balloon angioplasty (RA+BA).
BA = plain balloon angioplasty; MB = modified balloon; RA = rotational atherectomy.

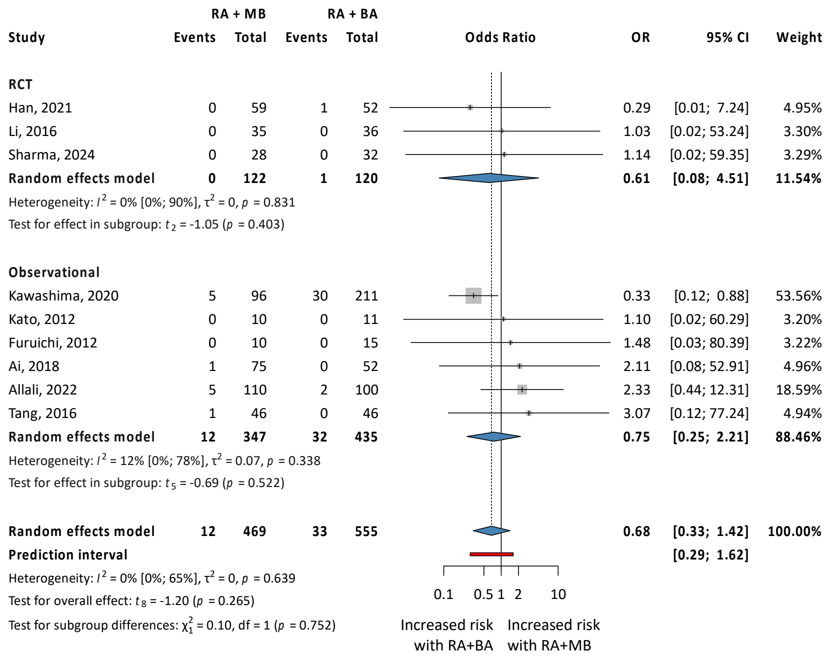

Supplementary Figure S2. **Results of the analysis of TLR rates.** Forest plots presenting the analysis of the target lesion revascularization (TLR) rate for the two groups treated with rotational atherectomy combined with either modified balloon types (RA+MB) or with plain balloon angioplasty (RA+BA).
BA = plain balloon angioplasty; MB = modified balloon; RA = rotational atherectomy.

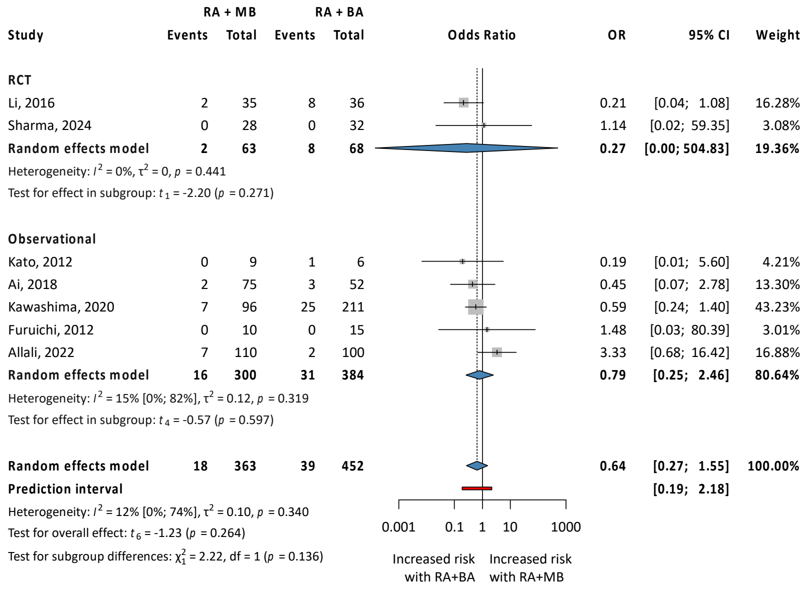

Supplementary Figure S3. **Results of the analysis of ACS rates.** Forest plot representing the analysis of the ACS rate for the two groups treated with rotational atherectomy combined with either modified balloon types (RA+MB) or with plain balloon angioplasty (RA+BA).

BA = plain balloon angioplasty; MB = modified balloon; RA = rotational atherectomy.

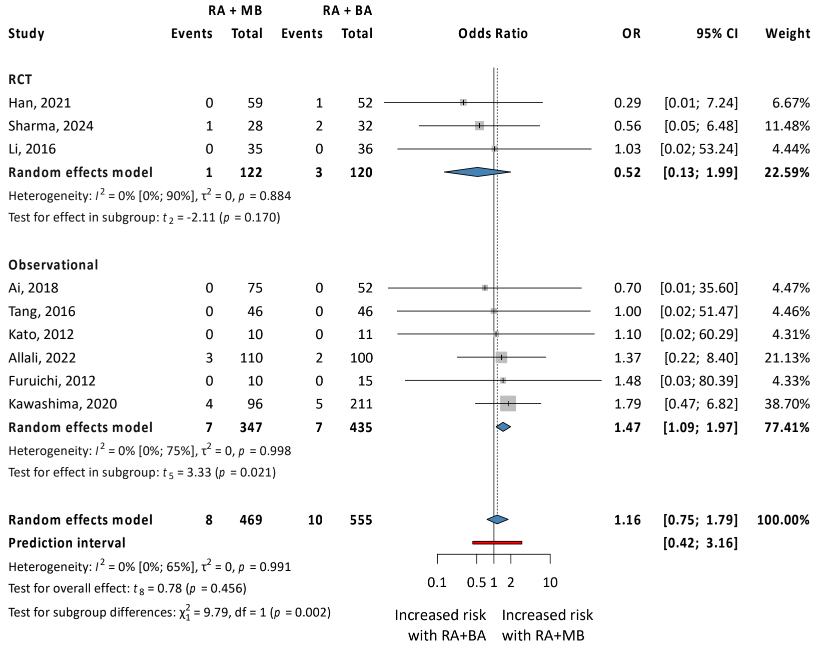

Supplementary Figure S4. **Results of the analysis of stent thrombosis rates.** Forest plot representing the analysis of the stent thrombosis rate for the two groups treated with rotational atherectomy combined with either modified balloon types (RA+MB) or with plain balloon angioplasty (RA+BA).

BA = plain balloon angioplasty; MB = modified balloon; RA = rotational atherectomy.

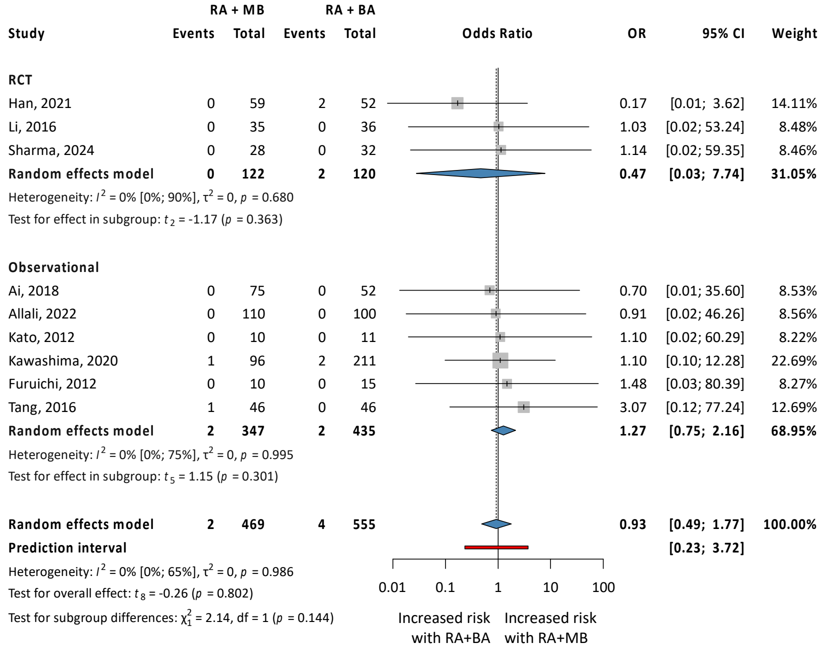

Supplementary Figure S5. Results of the analysis of TVR rates. Forest plot representing the analysis of the target vessel revascularization (TVR) rate for the two groups treated with rotational atherectomy combined with either modified balloon types (RA+MB) or with plain balloon angioplasty (RA+BA).
BA = plain balloon angioplasty; MB = modified balloon; RA = rotational atherectomy.

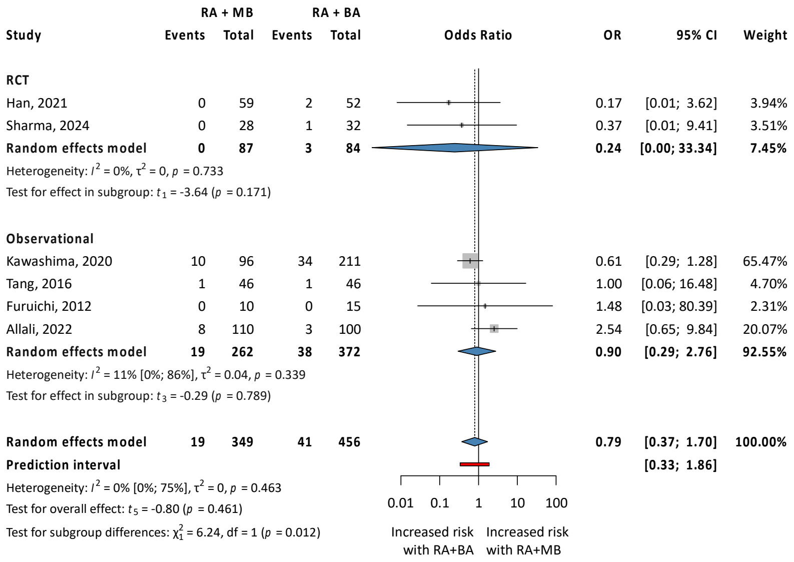

Supplementary Figure S6. Baujat plot. Baujat plot to explore the source of heterogeneity in the analysis of MACE.

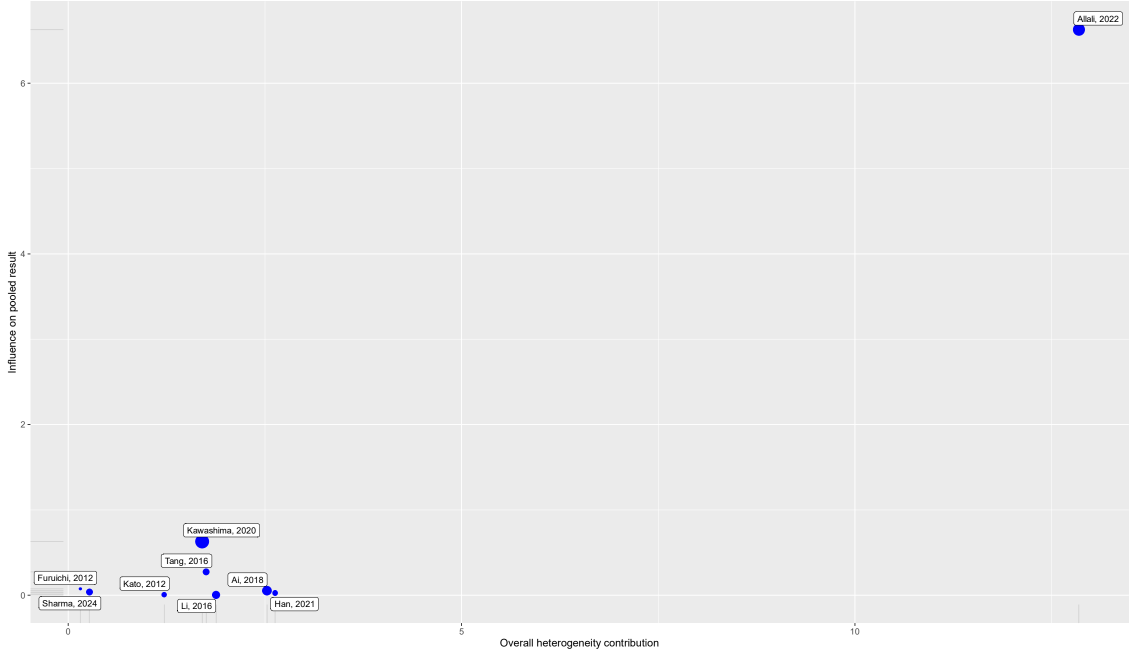

Supplementary Figure S7. Results of the Analyses of Safety Outcomes. ***Left:*** Forest plots presenting the analysis of slow-flow/no-reflow (***upper plot***) and coronary perforation (***lower plot***) rate for the two groups treated with rotational atherectomy combined with either modified balloon types (RA+MB) or with plain balloon angioplasty (RA+BA).
BA = plain balloon angioplasty; MB = modified balloon; RA = rotational atherectomy.

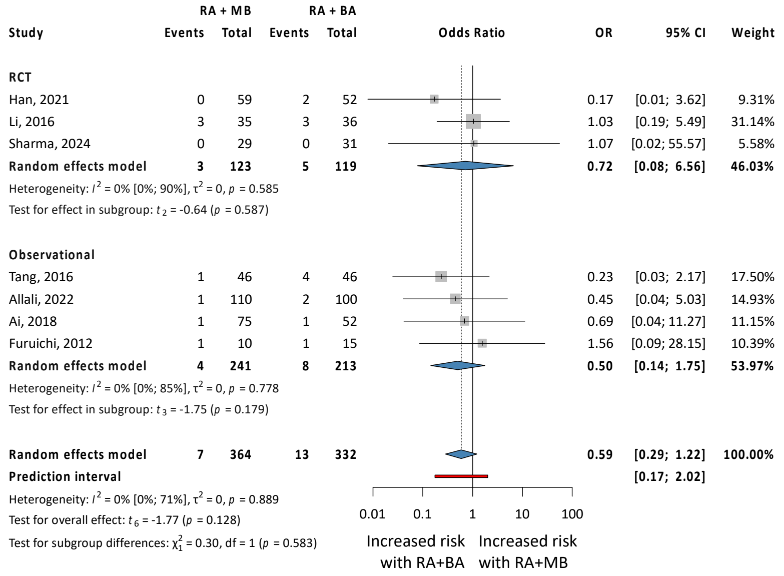

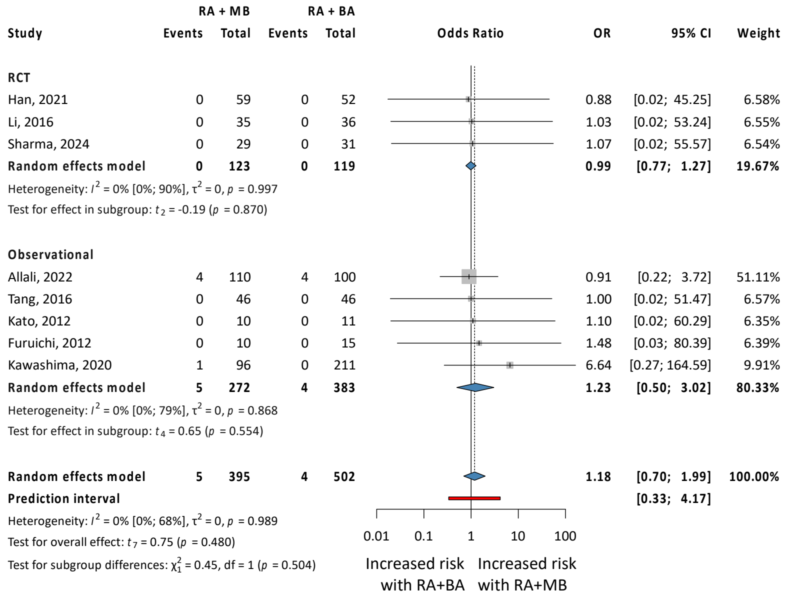

RISK OF BIAS AND CERTAINTY OF EVIDENCE ASSESSMENT RESULTS

Supplementary Figure S8. Risk of bias assessment for MACE using RoB2. Risk of bias assessment of outcome MACE and its components using the RoB2 tool for randomized studies.

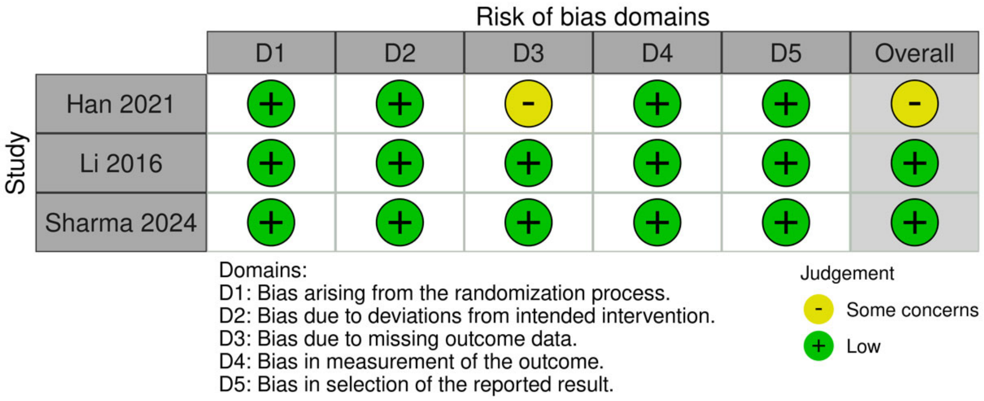

Supplementary Figure S9. Risk of bias assessment for MACE using ROBINS-I. Risk of bias assessment of outcome MACE and its components using the ROBINS-I tool for non-randomized studies.

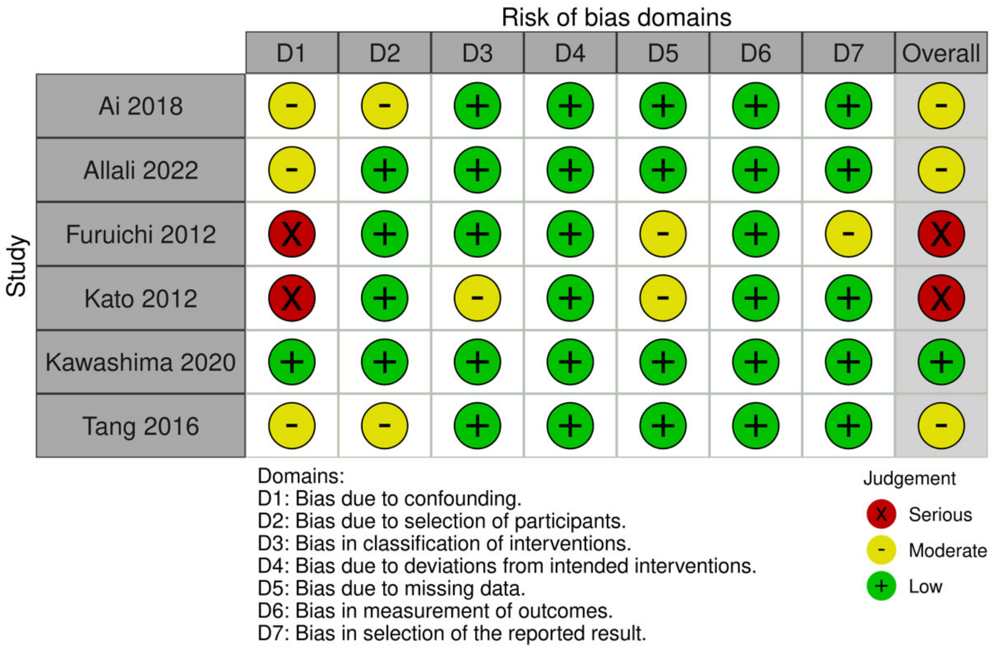

Supplementary Figure S10. Funnel plot for publication bias.

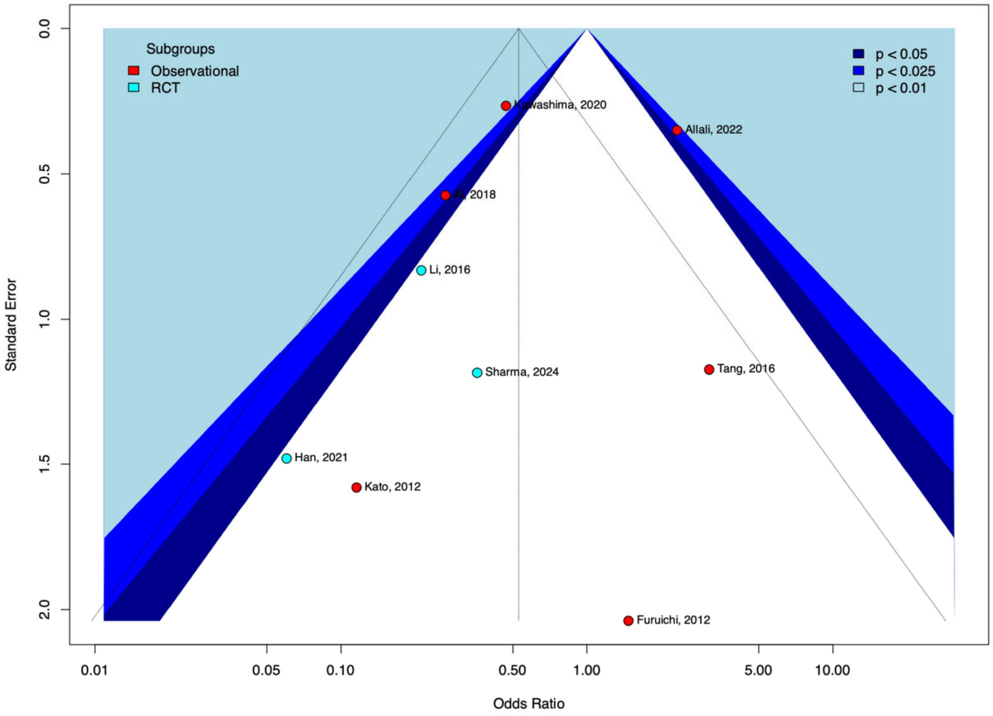

2 Supplementary Tables

Supplementary Table S1. PRISMA 2020 checklist.

Section and Topic	Item #	Checklist item	Location where item is reported
**TITLE**			
Title	1	Identify the report as a systematic review.	Title
**ABSTRACT**			
Abstract	2	See the PRISMA 2020 for Abstracts checklist.	Abstract
**INTRODUCTION**			
Rationale	3	Describe the rationale for the review in the context of existing knowledge.	Section 1
Objectives	4	Provide an explicit statement of the objective(s) or question(s) the review addresses.	Section 1
**METHODS**			
Eligibility criteria	5	Specify the inclusion and exclusion criteria for the review and how studies were grouped for the syntheses.	Section 2.1
Information sources	6	Specify all databases, registers, websites, organisations, reference lists and other sources searched or consulted to identify studies. Specify the date when each source was last searched or consulted.	Section 2.2
Search strategy	7	Present the full search strategies for all databases, registers and websites, including any filters and limits used.	Section 2.3+Suppl.
Selection process	8	Specify the methods used to decide whether a study met the inclusion criteria of the review, including how many reviewers screened each record and each report retrieved, whether they worked independently, and if applicable, details of automation tools used in the process.	Section 2.4+Suppl.
Data collection process	9	Specify the methods used to collect data from reports, including how many reviewers collected data from each report, whether they worked independently, any processes for obtaining or confirming data from study investigators, and if applicable, details of automation tools used in the process.	Section 2.5
Data items	10a	List and define all outcomes for which data were sought. Specify whether all results that were compatible with each outcome domain in each study were sought (e.g. for all measures, time points, analyses), and if not, the methods used to decide which results to collect.	Section 2.5
	10b	List and define all other variables for which data were sought (e.g. participant and intervention characteristics, funding sources). Describe any assumptions made about any missing or unclear information.	Section 2.5
Study risk of bias assessment	11	Specify the methods used to assess risk of bias in the included studies, including details of the tool(s) used, how many reviewers assessed each study and whether they worked independently, and if applicable, details of automation tools used in the process.	Section 2.6
Effect measures	12	Specify for each outcome the effect measure(s) (e.g. risk ratio, mean difference) used in the synthesis or presentation of results.	Section 2.7
Synthesis methods	13a	Describe the processes used to decide which studies were eligible for each synthesis (e.g. tabulating the study intervention characteristics and comparing against the planned groups for each synthesis (item #5)).	Section 2.1, 2.4, 2.5, 2.7
	13b	Describe any methods required to prepare the data for presentation or synthesis, such as handling of missing summary statistics, or data	Section 2.7

Section and Topic	Item #	Checklist item	Location where item is reported
		conversions.	
	13c	Describe any methods used to tabulate or visually display results of individual studies and syntheses.	Section 2.7
	13d	Describe any methods used to synthesize results and provide a rationale for the choice(s). If meta-analysis was performed, describe the model(s), method(s) to identify the presence and extent of statistical heterogeneity, and software package(s) used.	Section 2.7
	13e	Describe any methods used to explore possible causes of heterogeneity among study results (e.g. subgroup analysis, meta-regression).	Section 2.7
	13f	Describe any sensitivity analyses conducted to assess robustness of the synthesized results.	Section 2.7
Reporting bias assessment	14	Describe any methods used to assess risk of bias due to missing results in a synthesis (arising from reporting biases).	Section 3.6+Suppl.
Certainty assessment	15	Describe any methods used to assess certainty (or confidence) in the body of evidence for an outcome.	Section 2.6
**RESULTS**			
Study selection	16a	Describe the results of the search and selection process, from the number of records identified in the search to the number of studies included in the review, ideally using a flow diagram.	Section 3.1
	16b	Cite studies that might appear to meet the inclusion criteria, but which were excluded, and explain why they were excluded.	NA
Study characteristics	17	Cite each included study and present its characteristics.	Section 3.2
Risk of bias in studies	18	Present assessments of risk of bias for each included study.	Section 3.6+Suppl.
Results of individual studies	19	For all outcomes, present, for each study: (a) summary statistics for each group (where appropriate) and (b) an effect estimate and its precision (e.g. confidence/credible interval), ideally using structured tables or plots.	Section 3.3-3.5+Suppl.
Results of syntheses	20a	For each synthesis, briefly summarise the characteristics and risk of bias among contributing studies.	Section 3.6+Suppl.
	20b	Present results of all statistical syntheses conducted. If meta-analysis was done, present for each the summary estimate and its precision (e.g. confidence/credible interval) and measures of statistical heterogeneity. If comparing groups, describe the direction of the effect.	Section 3.3-3.5+Suppl.
	20c	Present results of all investigations of possible causes of heterogeneity among study results.	Section 3.3.1, 3.5+Suppl.
	20d	Present results of all sensitivity analyses conducted to assess the robustness of the synthesized results.	Section 3.3.1, 3.5+Suppl.
Reporting biases	21	Present assessments of risk of bias due to missing results (arising from reporting biases) for each synthesis assessed.	Section 3.6+Suppl.
Certainty of evidence	22	Present assessments of certainty (or confidence) in the body of evidence for each outcome assessed.	Section 3.7+Suppl.

Section and Topic	Item #	Checklist item	Location where item is reported
**DISCUSSION**			
Discussion	23a	Provide a general interpretation of the results in the context of other evidence.	Section 4
	23b	Discuss any limitations of the evidence included in the review.	Section 4
	23c	Discuss any limitations of the review processes used.	Section 4
	23d	Discuss implications of the results for practice, policy, and future research.	Section 4
**OTHER INFORMATION**			
Registration and protocol	24a	Provide registration information for the review, including register name and registration number, or state that the review was not registered.	Section 2
	24b	Indicate where the review protocol can be accessed, or state that a protocol was not prepared.	Section 2
	24c	Describe and explain any amendments to information provided at registration or in the protocol.	Section 2
Support	25	Describe sources of financial or non-financial support for the review, and the role of the funders or sponsors in the review.	Sources of funding
Competing interests	26	Declare any competing interests of review authors.	Conflict of interest
Availability of data, code and other materials	27	Report which of the following are publicly available and where they can be found: template data collection forms; data extracted from included studies; data used for all analyses; analytic code; any other materials used in the review.	Disclosures

From: Page MJ, McKenzie JE, Bossuyt PM, Boutron I, Hoffmann TC, Mulrow CD, et al. The PRISMA 2020 statement: an updated guideline for reporting systematic reviews. BMJ 2021;372:n71. doi: 10.1136/bmj.n71

For more information, visit: http://www.prisma-statement.org/

Supplementary Table S2. Extended baseline and intervention characteristics.

Author, year	Co-morbidities, confounders				Modified balloon type	Target vessel				Lesion characteristics		
	Diabetes (%)	Hyper-tension (%)	Dys-lipidemia (%)	Smoking (%)		LM (%)	LAD (%)	LCX (%)	RCA (%)	Ostial (%)	Bifurcation (%)	CTO (%)
Furuichi, 2012[13]	ND	ND	ND	ND	cutting	0/ND	80/ND	10/ND	10/ND	ND/ND	ND/ND	ND/ND
Kato, 2012[30]	70/64	90/91	90/73	60/55	scoring	0/0	80/64	0/11	20/27	ND/ND	ND/ND	0/0
Tang, 2016[31]	35/37	80/85	26/17	24/46	cutting	0/0	89.1/76.1	6.5/15.2	4.3/8.7	ND/ND	10.9/15.2	ND/ND
Li, 2016[32]	71/75	77/78	51/56	57/61	cutting	22.9/5.6	57.1/63.9	14.3/11.1	20/25	8.6/11.1	37.1/44.4	0/0
Ai, 2018[33]	63/56	67/64	35/39	61/37*	cutting	0/0	68/75	9.3/5.8	22.7/19.2	9.3/11.5	57.3/61.5	ND/ND
Kawashima, 2020[16]	63/58	78/84	74/70	ND	scoring	8.3/9	69.8/64.9	6.3/10.9	15.6/14.2	ND/ND	ND/ND	5.2/6.6
Han, 2021[34]	37/33	45/38	52/58	47/52	cutting	11.7/8.3	51.7/56.7	5/6.7	31.7/28.3	11.7/15.3	41.7/35	ND/ND
Allali, 2022[35]	31/33	87/93	45/68*	17/15	cutting	11.3/10.6	43.1/55.3	12.5/11.3	33.1/22.7	29.4/28.4	49.4/39	3.8/2.8
Sharma, 2024[36]	41/42	97/94	97/94	11/19	cutting	0/ND	62.1/51.6	13.8/9.7	24.1/38.7	ND/ND	ND/ND	ND/ND

CTO = chronic total occlusion; LAD = left anterior descending coronary artery; LCX = left circumflex coronary artery; LM = left main coronary artery; ND=no data available; RCA = right coronary artery.

Supplementary Table S3. Certainty of evidence assessment of the individual outcomes using GRADE-Pro.

Certainty assessment							№ of patients		Effect		Certainty	Importance
№ of studies	Study design	Risk of bias	Inconsistency	Indirectness	Imprecision	Other considerations	RA + MB	RA + BA	Relative (95% CI)	Absolute (95% CI)		
**Major adverse cardiovascular events**												
9	non-randomised studies[a]	serious[b]	serious[c]	not serious	not serious	none	70/469 (14.9%)	143/555 (25.8%)	**OR 0.53** (0.21 to 1.34)	**102 fewer per 1 000** (from 190 fewer to 60 more)	⊕⊕◯◯ Low[b,c]	CRITICAL
**Acute coronary syndrome**												
9	non-randomised studies	serious[b]	not serious	not serious	very serious[d]	none	8/469 (1.7%)	10/555 (1.8%)	**OR 1.16** (0.75 to 1.79)	**3 more per 1 000** (from 4 fewer to 14 more)	⊕◯◯◯ Very low[b,d]	CRITICAL
**All-cause death**												
9	non-randomised studies	serious[b]	not serious	not serious	serious[d]	none	12/469 (2.6%)	33/555 (5.9%)	**OR 0.68** (0.33 to 1.42)	**18 fewer per 1 000** (from 39 fewer to 23 more)	⊕⊕◯◯ Low[b,d]	CRITICAL
**Target lesion revascularization**												
7	non-randomised studies	serious[b]	not serious	not serious	serious[d]	none	18/363 (5.0%)	39/452 (8.6%)	**OR 0.64** (0.27 to 1.55)	**29 fewer per 1 000** (from 61 fewer to 41 more)	⊕⊕◯◯ Low[b,d]	IMPORTANT
**Target vessel revascularization**												
6	non-randomised studies	serious[b]	not serious	not serious	serious[e]	none	19/349 (5.4%)	41/456 (9.0%)	**OR 0.79** (0.37 to 1.70)	**18 fewer per 1 000** (from 55 fewer to 54 more)	⊕⊕◯◯ Low[b,e]	IMPORTANT
**Stent thrombosis**												
9	non-randomised studies	serious[b]	not serious	not serious	very serious[d]	none	2/469 (0.4%)	4/555 (0.7%)	**OR 0.93** (0.49 to 1.77)	**1 fewer per 1 000** (from 4 fewer to 5 more)	⊕◯◯◯ Very low[b,d]	IMPORTANT

Certainty assessment							№ of patients		Effect		Certainty	Importance
№ of studies	Study design	Risk of bias	Inconsistency	Indirectness	Imprecision	Other considerations	RA + MB	RA + BA	Relative (95% CI)	Absolute (95% CI)		
**No-flow/slow-flow**												
7	non-randomised studies	serious[b]	not serious	not serious	serious[d]	none	7/364 (1.9%)	13/332 (3.9%)	**OR 0.59** (0.29 to 1.22)	**16 fewer per 1 000** (from 27 fewer to 8 more)	⨁⨁◯◯ Low[b,d]	IMPORTANT
**Coronary perforation**												
8	non-randomised studies	serious[b]	not serious	not serious	serious[d]	none	5/395 (1.3%)	4/502 (0.8%)	**OR 1.18** (0.70 to 1.99)	**1 more per 1 000** (from 2 fewer to 8 more)	⨁⨁◯◯ Low[b,d]	IMPORTANT
